# Efficacy of T-Cell Receptor-Based Adoptive Cell Therapy in Cutaneous Melanoma: A Meta-Analysis

**DOI:** 10.1093/oncolo/oyad078

**Published:** 2023-04-10

**Authors:** Ramon Yarza, Mateo Bover, Mercedes Herrera-Juarez, Macarena Rey-Cardenas, Luis Paz-Ares, Jose A Lopez-Martin, John Haanen

**Affiliations:** Department of Medical Oncology, Hospital Universitario 12 de Octubre, Madrid, Spain; Department of Medical Oncology, Hospital Universitario 12 de Octubre, Madrid, Spain; Department of Medical Oncology, Hospital Universitario 12 de Octubre, Madrid, Spain; Department of Medical Oncology, Hospital Universitario 12 de Octubre, Madrid, Spain; Department of Medical Oncology, Hospital Universitario 12 de Octubre, Madrid, Spain; Virology and Inflammation Unit, PharmaMar, SA, Madrid, Spain; GETICA (Spanish Group for Cancer Immuno-Biotherapies), Madrid, Spain; Division of Medical Oncology, Netherlands Cancer Institute, Amsterdam, The Netherlands

**Keywords:** adoptive cell therapy, T-cell receptor, cutaneous melanoma, meta-analysis

## Abstract

**Background:**

T-cell receptor (TCR-T) therapies are based on the expression of an introduced TCR targeting a tumor associated antigen (TAA) which has been studied in several trials in cutaneous melanoma. We conducted a systematic review and meta-analysis aiming to assess the primary efficacy of TCR-based adoptive cell therapy in cutaneous melanoma.

**Methods:**

We searched through PubMed electronic database from its inception until May 21, 2022. Primary endpoints were pooled objective response rate (ORR) and disease control rate (DCR). We conducted logistic regression analyses to identify potential predictive factors for tumor response.

**Results:**

From 187 patients, 50 showed an objective response (pooled ORR 28%; 95% CI, 20%-37%) and a pooled DCR of 38% (95% CI, 27%-50%). Median PFS was 2, 9 months (95% CI, 1.4-3.1). A trend toward higher PFS was demonstrated for patients treated with cancer/testis antigens targeting TCR-T cells (HR 0.91 95% CI, 0.64-1.3, *P* = .61) among whom, patients treated with NYESO-1 targeting TCR-T showed a significantly higher PFS (HR 0.63 95% CI, 0.64-0.98, *P* = .03). In addition, the number of infused cells was associated with a significantly higher likelihood of tumor response (OR 6.61; 95% CI, 1.68-21.6; *P* = .007).

**Conclusion:**

TCR-T therapy shows promising results in terms of antitumor activity and survival similar to those reported for TILs with a significantly higher benefit for cancer/testis antigens targeting cells. Since TCR-based therapy shows advantages of great potential over classic ACT strategies, further research in solid cancers is warranted (PROSPERO ID CRD42022328011).

## Teaser

Adoptive Cell Therapy represents a novel therapeutic modality in cancer. To this extent, TCR based ACT modelling has become a suitable strategy with the aim of targeting specific tumour associated antigens. This works provides a pool metanalysis of the efficacy and antitumor activity of TCR based ACT experience in metastatic cutaneous melanoma.

**Implications for Practice**

Adoptive cell therapy aims to target cancer cells by genetically engineered immune cells with the objective of enhancing tumor recognition and effective tumor killing. We demonstrate that TCR-T-based ACT has similar efficacy results compared to those reported for TILs among heavily pretreated melanoma patients. We also found that NYESO-1 targeting TCR-T cells show significantly longer PFS and higher ORR. Furthermore, our results revealed that TCR-T efficacy is dose-dependent, with a significantly higher response probability associated with the infused cell product. These results are of the highest interest since they represent novel therapeutic perspectives in cancer even among heavily pretreated patients.

## Introduction

Tumor immunology has yielded an unprecedented hallmark in cancer demonstrating the immunogenicity of some cancers, which could be exploited by harnessing the immune system to recognize and kill cancer cells. Adoptive cell therapy (ACT) represents one such immunotherapeutic modality utilizing immune cells with specific tumor-recognizing receptors aimed to display on-tumor toxicity.^1^

ACT has been the subject of intense research in the past decades culminating in a wide variety of cell-engineering models that have yielded diverse possibilities for targeting tumor cells.^2^ Since identification of tumor-infiltrating immune cells in the 1980s,^3^ Rosenberg et al worked on the purification and expansion of tumor infiltrating lymphocytes (TILs) prompting cellular recovery from tumor-induced inhibition by culturing cells in interleukin 2 (IL2) enriched media.^4^ ACT can be classified into 3 different groups with notable differences in terms of specificity, clonality, and target recognition: TILs, T-cell receptor engineered immune cells (TCR-T), and chimeric antigen receptor expressing cells (CAR-T).^5^ TILs represent a strategy based on the ex vivo purification and expansion of polyclonal tumor-specific lymphocytes from the tumor nest. In essence, TILs exert on-tumor toxicity since their activation is dependent on the presence of previously recognized tumor associated antigens (TAA). However, the main limitations of TILs reside in the heterogeneity of the infusion product whose major histocompatibility complex (MHC) restriction and antigen-specificity remain complex to elucidate.^1^ In contrast, CAR therapy is based on genetic modification of tumor-­infiltrating or peripheral blood immune cells to express membrane receptors composed of a single-chain variable fraction (scFv) with further addition of intracellular signaling domains of co-stimulatory molecules. CAR-T can recognize surface antigens in an MHC unrestricted fashion. Nevertheless, the time-­limited persistence of circulating CAR-T cells still represents the main limitation of this strategy, which has been widely proven to be inversely correlated with a sustained tumor response.^6^

TCR-T are either modified tumor-residing (TIL) or peripheral blood immune cells that harbor a genetically induced and antigen-specific TCR. Expression of this construct results in a functional supramolecular activation cluster (SMAC) bearing the genetically encoded TCRαβ together with the endogenous CD3 complex molecules (CD3 γ, δ, ε, ζ chains) as well as active co-receptors (CD4 and CD8) and co-stimulators (CD28 and 4-BB1).^7^ Therefore, TCR-based strategies exert antigen-specific tumor toxicity with the eventual generation of lasting antitumor memory.^1^

TCR-T based strategies have been preliminarily exploited in both solid and hematologic malignancies.^7^ In this regard, malignant melanoma has been a role model because of its highly immunogenic nature.^8^ Moreover, prior identified TCR targetable TAAs include melanoma differentiation antigens, such as MART1, cancer/testis antigens including NYESO and the MAGE-family proteins, as well as viral antigens.^1^ Up to 80%-95% of melanomas express MART1.^9^ Similarly, NYESO and MAGE-A3 have been described to be present in approximately 52%^10^ and 62%^11^of melanomas. These shared TAAs presented by HLA-A*0201 which is present in approximately 45% of the Caucasian population. Given the high incidence of TAA expression, TCR therapy has been widely studied in melanoma supporting its suitability in the setting of this disease.^7^

Given the fragmented evidence on the efficacy of TCR-T therapies which derives from early phase trials, we conducted a systematic review and meta-analysis to assess the preliminary efficacy of TCR-based ACT in cutaneous melanoma.

## Material and Methods

We used the preferred reporting items for systematic reviews and meta-analyses (PRISMA) guidelines to ensure the quality of the study. Our work was registered in the International prospective register of systematic reviews of the National Institute for Health Research (NIHR, PROSPERO CRD42022328011).

### Eligibility Criteria

We searched for both randomized and non-randomized studies using TCR-based ACT models regardless of interleukin 2 (IL2) and/or non-myeloablative (NMA) lymphodepleting chemotherapy administration. Studies were further divided into different groups according to pre-specified IL2 dosage as follows: studies administering standard intravenous IL2 at 600,000-720,000 IU/kg every 8 h to tolerance were categorized as high dose IL2 (HD); those administering any lower dose of IL2 were categorized as low dose IL2 (LD); finally, we included a third group for those studies not administering IL2. Similarly, studies were further categorized in NMA administering and NMA absent groups. Target population for the study included patients diagnosed with malignant melanoma regardless of clinical stage. Both, studies including pretreated patients as well as treatment naïve individuals were eligible for final analysis. Tumor response had to be individually reported to further assess primary and secondary endpoints, which was further assessed using RECIST criteria for tumor response assessment. Exclusion criteria included uveal/mucosal melanoma, any other ACT model than TCR, and combinatorial strategies such as combined DC vaccination as well as single-case reports.

### Search Strategies and Study Selection

**Figure 1.**
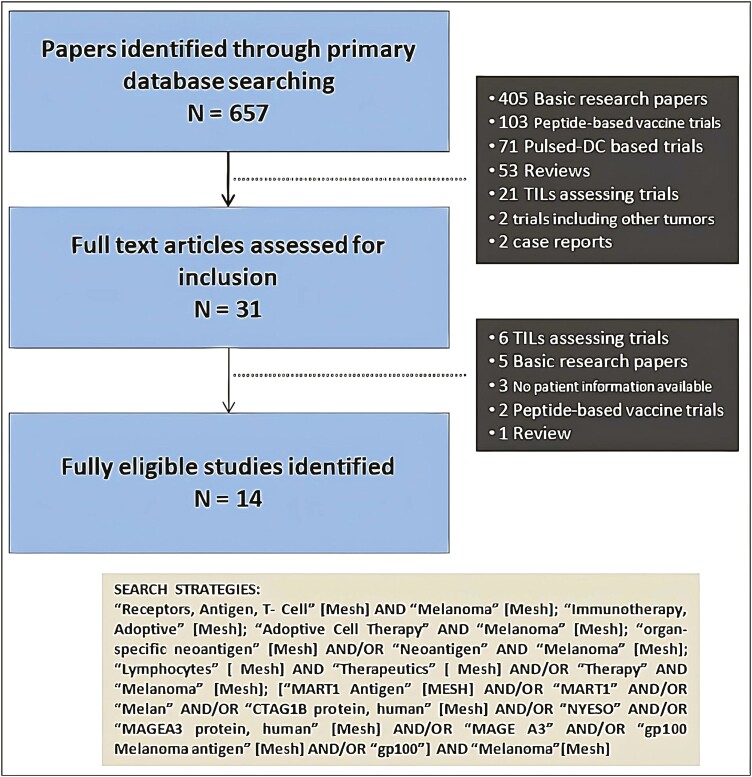
Flow chart of study selection.

We searched through the PubMed electronic database for studies that accomplished all the above-specified criteria without date or language restriction, including all studies published since inception until the date in which the search took place. The search was performed on May 21, 2022. The search strategy as well as the corresponding study selection flow chart is shown in Fig. 1.

We used a stepwise method to ensure an accurate search. First, eligibility assessment was independently performed by 3 (RY, MB, and MH) reviewers who were responsible for both title and abstract review from which a preliminary eligibility screening was carried out. Second, full text review of previously selected studies was performed. Any discordance on study selection was further discussed among all authors and was eventually solved by consultation with specialists on ACT (JH). Double-counting was controlled and avoided adjusting by author’s name, sample size, and recruitment period. Prior to search, we decided to include only data from most recent publications in case of double-counting description cohorts, although we did not encounter such a case during the data extraction process. All studies included were finally reviewed by all authors and discussed separately.

### Data Extraction Process

Data extraction was conducted independently by 2 reviewers (RY, MH) and cross-checked by a third (MB) following the pre-specified protocol for variables of interest. All data collected were available at the individual level, which was eventually used for pool analysis. Information from the data extracted is shown in Table 1.

**Table 1.** Characteristics of the patients included in the study. Overall pool estimates of individual data available for each patient.

	*N*	Sex		Age	Previous therapies received				Stage					BM	Num. of cells infused (*10^9^)		ORR	DCR	PFS		OS	
		Male	Female	Mean	Naïve	CHT	ICIs	BioCHT	IIIB	IIIC	M1a	M1b	M1c		Mean	SD			Median	CI 95%	Median	CI 95%
Chodon T et al 2014^12^	13	61% (8)	39% (5)	51.3 (2)	54% (7)	8% (1)	23% (3)	38% (5)	0	0	8% (1)	31% (4)	62% (8)	0	2.03	0.43	0	54% (7)	3	2.1-4.1	8	4.1-11
Dréno B et al 2021^13^	6	34% (2)	66% (4)	65.5 (5.3)	66% (4)	0	33% (2)	33% (2)	0	83% (5)	0	17% (1)	0	0	0.78	0.11	17% (1)	17% (1)	3.1	1.7-5.3	—	—
Duval L et al 2015^14^	15	53% (8)	47% (7)	41.1 (2.9)	80% (12)	0	0	20% (3)	0	0	27% (4)	27% (4)	46% (7)	0	0.68	0.18	20% (3)	20% (3)	NR	NR	6.7	NR*
Fontana R et al 2009^15^	9	NR	NR	NR	0	67% (6)	11% (1)^++^	78% (7)	0	11% (1)	0	22% (3)	55% (5)	0	0.34	0.06	11% (1)	22% (2)	1	0.8-9.1	—	—
Johnson LA et al 2009/1^16^	20	55% (11)	45% (9)	46.3 (2.4)	0	25% (5)	0	100% (20)	0	0	30% (6)	10% (2)	45% (9)	15% (3)	26.4	6.6	30% (6)	30% (6)	1	0.9-3.3	—	—
Johnson LA et al 2009/2^16^	16	56% (9)	44% (7)	44.2 (2.7)	0	50% (8)	0	100% (16)	0	0	31% (5)	13% (2)	44% (7)	13% (2)	30.1	8.7	19% (3)	19% (3)	0.9	0.9-1	—	—
Khammari A et al 2009^17^	14	36% (5)	64% (9)	63.3 (3.2)	0	100% (14)	0	0	43% (6)	28% (4)	21% (3)	0	7% (1)	0	0.9	0.2	43% (6)	50% (7)	3	2.9-6	—	—
Lu YC et al 2017^18^	6	83% (5)	17% (1)	51.9 (4.3)	0	33% (2)	100% (6)	66% (4)	0	0	17% (1)	17% (1)	66% (4)	0	19.1	14.4	0	0	0.9	0.8-1	—	—
Mackensen A et al 2006^19^	11	45% (5)	55% (6)	53.2 (3.3)	0	100% (11)	72% (8)^+^	27% (3)	0	0	54% (6)	18% (2)	27% (3)	0	0.97	0.29	27% (3)	36% (4)	1.4	1.3-11	—	—
Mitchell MS et al 2002^20^	10	NR	NR	NR	0	100% (10)	0	0	0	0	20% (2)	10% (1)	40% (4)	30% (3)	0.44	0.05	10% (1)	10% (1)	3	1.2-3.5	5.6	1-15.1
Morgan RA et al 2006^21^	17	65% (11)	35% (6)	42.4 (2.9)	NR	NR	NR	100% (17)	0	0	18% (3)	59% (10)	18% (3)	6% (1)	16.4	5	12% (2)	12% (2)	21	0.2-10.6	—	—
Morgan RA et al 2013^22^	9	44% (4)	56% (9)	51.9 (5.1)	0	78% (7)	0	89% (8)	0	0	11% (1)	22% (2)	44% (4)	22% (2)	46	6.1	56% (5)	56% (5)	4	0-4.5	—	—
Robbins PF et al 2011^23^	20	75% (15)	25% (5)	48.3 (2.5)	5% (1)	32% (6)	0	95% (19)	0	0	35% (7)	15% (3)	45% (9)	5% (1)	50.5	6	55% (11)	55% (11)	3.3	1.3-10.1	—	—
Robbins PF et al 2015^24^	11	64% (7)	36% (4)	46.5 (3.2)	0	36% (4)	0	100% (11)	0	0	27% (3)	18% (2)	45% (5)	9% (1)	50.7	9.3	45% (5)	45% (5)	1.4	1.7-11	—	—
Yee C et al 2002/1^25^	5	0% (0)	100% (5)	48.8 (2)	0	60% (3)	0	60% (3)	0	0	40% (2)	0	60% (3)	0	ND	ND	40% (2)	80% (4)	15.2	3.2-18.9	—	—
Yee C et al 2002/2^25^	5	60% (3)	40% (2)	51 (3.6)	0	0	0	100% (5)	0	0	0	60% (3)	40% (2)	0	ND	ND	20% (1)	80% (4)	6.8	2.1-11.1	—	—
Overall	187	52% (93)	42% (74)	49.9 (0.9)	13% (24)	41% (77)	11% (20)	65% (123)	3% (6)	5% (10)	23% (44)	22% (40)	39% (74)	7% (13)	3.9^**^	2.1	27% (50)	35% (65)	3	1.4-3	NA	NA

Percentage expressed as % (*n*).

BioCHT, biochemotherapy including IL2 and/or interferon; BM, brain metastases; CHT, chemotherapy; ICIs, immune checkpoint inhibitors; *N*, sample size; NA, non-applicable; ND, non-deductible NR, not reported.

NR* (reported IQR 1-25).

^**^Overall number of cells expressed as median.

^+^Specified as “immunotherapy” by the authors without specific mention of ICI.

^++^Specified as “no IL2, no IFN based idiotype monoclonal antibody” by the authors without specific mention of ICI.

### Statistical Analyses

The primary endpoints included objective response rate (ORR) and disease control rate (DCR). Secondary endpoints were progression free survival (PFS) and overall survival (OS). We also aimed to look for clinical predictive markers for tumor response according to extracted variables. These variables included age, sex, tumor stage, prior therapies, number of infused cells, IL2 administration, and NMA lymphodepletion. Data were collected as quantitative continuous when possible. We built qualitative, either dichotomous (lymphodepletion, previous treatment) or polychotomous variables (tumor stage, IL2 administration). For further categorization of quantitative variables into binary expression, we used a cutoff of up to 50 years in the case of age, and we used median-based cutoff for number of infused cells. Survival time was collected according to data in the articles accomplishing all eligibility criteria. For those studies reporting no exact survival time in the case of progressive disease (PD) patients, we considered PFS to be the time from therapy initiation until the first reevaluation according to the study protocol. Descriptive analyses are expressed as percentages with a 95% confidence interval (CI). For quantitative variables, we used means together with 95% CI.

We used either Maentel-Haenszel fixed model (FEM) or DerSimonian-Laird random model (REM) for calculation of pooled estimates depending on a pre-specified level of significant heterogeneity (*P* > .1 in Cochran’s *Q* test for FEM election). Heterogeneity was assessed using Cochran’s *Q* and Higgins *I*^2^. We also performed different subgroup analyses. Publication bias was eventually assessed using Egger’s regression and funnel plotting.

Survival assessment was carried out by dropping Kaplan-Meier curves for PFS as well as for duration of response (DoR) and duration of stability (DoS). Existing hazard differences were assessed via the log-rank test at a pre-specified 2-sided *α* = 0.05 level of statistical significance and hazard ratios were estimated using Cox proportional regression model.

Finally, we sought predictive variables of tumor response using binary logistic regression models with further adjustment for potential confounders. We expressed odds ratios (OR) together with their corresponding 95% CI. Analysis was driven at a pre-specified 2-sided alpha error of 0.05 and statistical significance was accepted at any *P* < .05. We used STATA v16.1 (College Station, TX, StataCorp LLC) for statistical analysis.

## Results

### Studies Included

Primary search screened a total of 657 articles from which 31 were deemed for full review analysis. Out of these, 14 studies^12–25^ were finally identified that met all the predefined inclusion criteria. The search strategy, study identification and selection process are underlined in Fig. 1. Each of study characteristics are shown in Supplementary Material S1. Funnel plot for ORR showed symmetrical distribution with a non-significant Egger’s regression test (*P* = .12)—Supplementary Material S2.

### Cohorts

A total of 190 patients were identified from which target variables were extractable at an individual level. Three patients were not included in the final analysis for the following reasons: Chodon et al reported no efficacy information on patient F5-5 which was eventually excluded; Fontana et al included one patient with no evident disease (NED) on accrual, from whom neither ORR nor PFS was evaluable; Lu et al included one patient with mucosal melanoma that was eventually excluded from analysis. Therefore, a total of 187 patients were finally available for the full analysis. Mean age of the whole cohort was 49.9 years. Only 13% of patients were treatment naïve, whereas up to 41% and 65% of patients had received either previous chemotherapy (CHT) or biochemotherapy (previous IL2 and/or interferon-based therapies), respectively. Up to 11% of patients had undergone previous immunotherapy based on single or dual immune checkpoint blockade (ICB) using programmed death 1 inhibitor (iPD1) and/or cytotoxic T-lymphocyte associated protein 4 inhibitors (iCTLA-4). In some cases, immunotherapy strategy was not otherwise specified as underlined in Table 1. In addition, 68% of patients had visceral involvement, and up to 7% of the whole cohort had been previously diagnosed with brain metastasis. All studies except for Duval et al^14^ reported PFS data, whereas overall survival was only identified in 3 studies, which was not further analyzed given the lack of sample size for any informative result. Both individually analyzed information for each study as well as for pooled analysis for the whole cohort are shown in Table 1.

### Objective Response Rate

**Figure 2.**
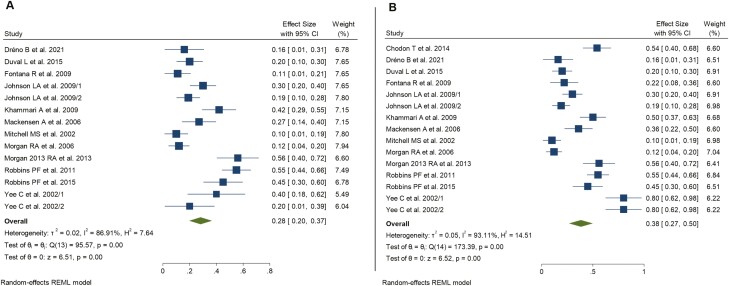
Forest plot for objective response rate (**A**) and disease control rate (**B**). Pool estimates of all studies included in the analysis.

**Figure 3.**
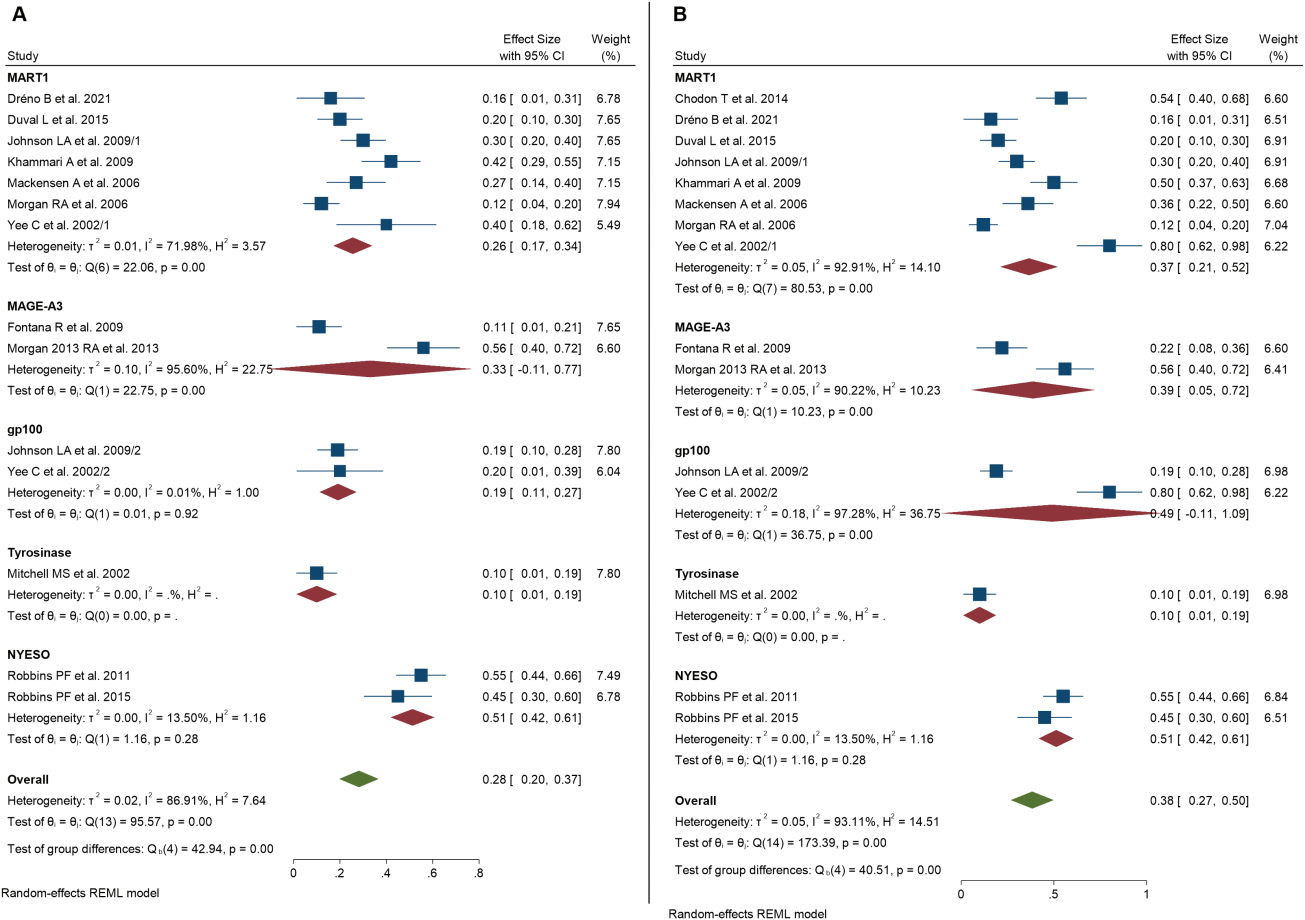
Subgroup analysis for each tumor-associated antigen (TAA). Forest plot for objective response rate (**A**) and disease control rate (**B**). Each subgroup is analyzed by REM. Overall estimates include pool estimates without subgrouping.

**Figure 4.**
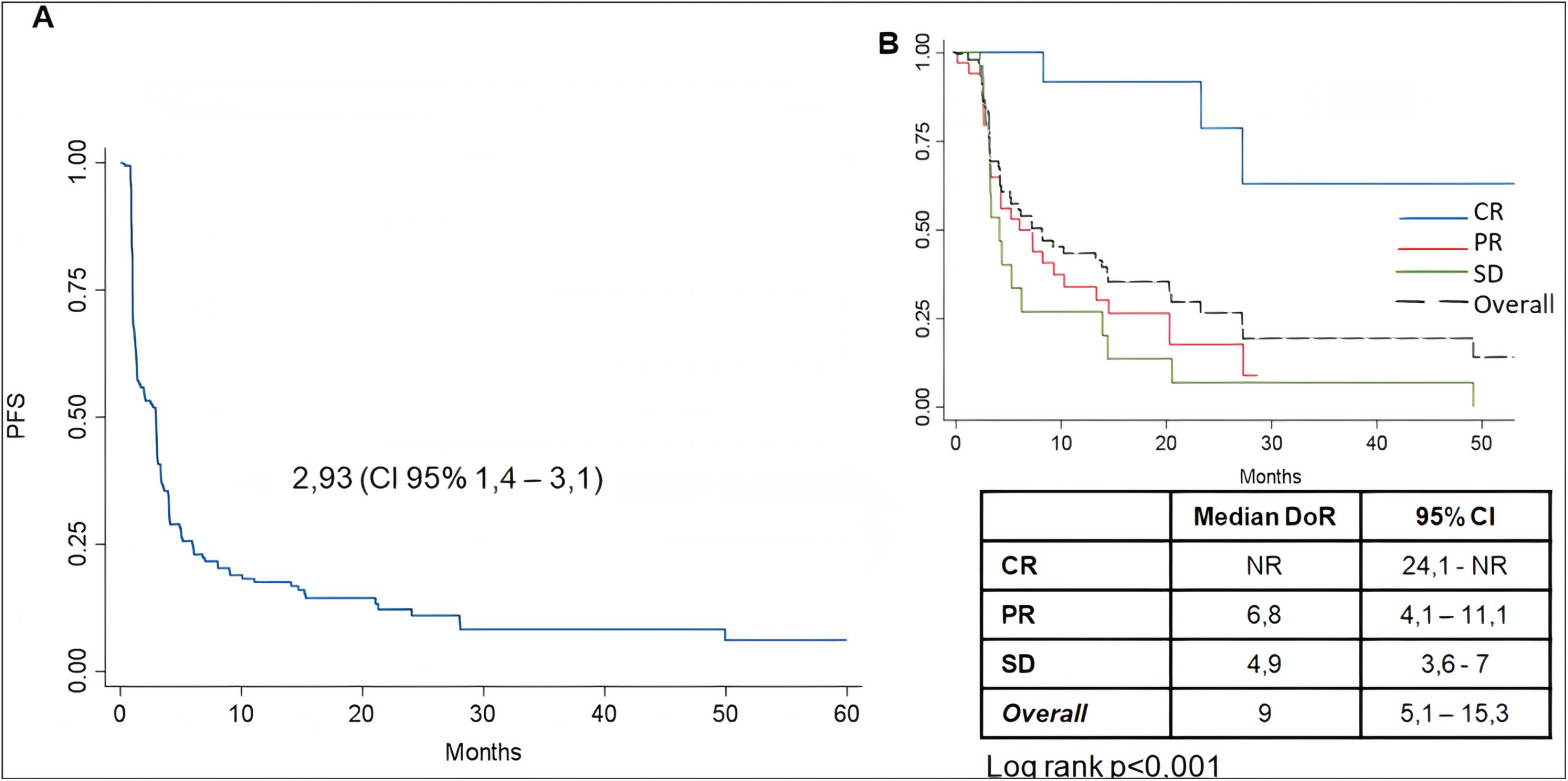
Kaplan-Meier plot for progression free survival (**A**) and duration of response/duration of stability (**B**). Table presents DoR/DoS results for each group with their 95% CIs. Abbreviations: CR, complete response; PR, partial response; SD, stable disease. Log-rank test for differences among CR vs. PR/SD.

The pooled overall ORR for the global population was 28% (*n* = 50/187 responses; CR *n* = 12, PR *n* = 38; 95% CI, 20%-37%; REM global *z* test *P* < .001; *I*^2^ = 86.9%, Fig. 2). After performing further subgroup analysis dividing the whole cohort by the IL2 dosage received, we found that ORR was 20% for the subgroup that did not receive post-infusional IL2 (95% CI, 6%-34%; *I*^2^ = 86.9%, Supplementary Material S3), 24% for patients undergoing LD IL2 (95% CI, 16%-32%; *I*^2^ = 4.36% Supplementary Material S3) and 35% for patients receiving HD IL2 (95% CI, 20%-51%; *I*^2^ = 91.7%, Fig. 4). Further subgroup analysis by lymphodepletion yielded an overall ORR of 22% among patients without NMA (95% CI, 14%-30%; *I*^2^ = 71.8%, Supplementary Material S4) and 35% among those receiving NMA (95% CI, 20%-51%; *I*^2^ = 91.7%, Supplementary Material S4). Matching afterward by targeted TAA yielded the highest ORR among patients treated with NYESO-1 targeting TCR modified T cells (pooled ORR 51%, 95% CI, 42%-61%; *I*^2^ = 13.5%, Fig. 3). Overall ORR for MART1 targeting TCR gene therapy reached 26% (95% CI, 17%-34%, *I*^2^ = 72%, Fig. 3); MAGE-A3 reached an ORR up to 33% (95% CI, 11%-77%; *I*^2^ = 95.6%, Fig. 3). Interestingly, sensitivity analyses including only trials using TCR-T specifically targeting cancer/testis antigens yielded higher ORR of up to 41% (95% CI, 20%-63%, *I*^2^ = 91.5%)—SuppLementary Material S5.

### Disease Control Rate

A total of 71 out of 187 patients were found to present either CR, PR, or SD during treatment (pooled overall DCR 38%, 95% CI, 27%-50%, REM global *z* test *P* < .001; *I*^2^ = 93.1%, Fig. 2). Subgroup analyses for IL2 adjuvant administration revealed a pooled DCR of 25% among patients who did not receive IL2 (95% CI, 28%-42%; *I*^2^ = 89.1%, SuppLementary Material S3), 53% in patients belonging to LD group (95% CI, 21%-84%; *I*^2^ = 96.6%, SuppLementary Material S3) and up to 38% among those receiving HD (95% CI, 24%-52%; *I*^2^ = 91.1%, SuppLementary Material S3). We found of notable interest that subsequent clustering for lymphodepletion yielded an overall DCR of 39% among patients without NMA (95% CI, 20%-58%; *I*^2^ = 94.4%, SuppLementary Material S4) which was similar to that for patients receiving prior NMA, who showed an overall DCR of 38% (95% CI, 24%-52%; *I*^2^ = 91.1%, SuppLementary Material S4). This fact remains of the highest interest since it may question the importance of NMA as a necessary step in TCR gene therapy. Finally, the analysis aiming to look for differences in the efficacy of TCR-modified T cells based on targeted TAA showed an overall DCR of 37% (95% CI, 21%-52%; *I*^2^ = 92.9%, Fig. 2), 39% (95% CI, 5%-72%; *I*^2^ = 90.2%, Fig. 2) and 51% (95% CI, 42%-61%; *I*^2^ = 13.5%, Fig. 2) for MART1, MAGE-A3, and NYESO, respectively. Similarly to what we found for ORR, sensitivity analysis for studies assessing the clinical efficacy of TCR T-cells targeting cancer/testis antigens yielded better outcomes in terms of DCR of up to 45% (95% CI, 29%-60%; *I*^2^ = 80.9%)—Supplementary Material S5.

### Survival Analysis: Progression-free Survival, Duration of Response and Duration of Stability

Median PFS, based on the available individual patient data for 12 studies, was 2.9 months (95% CI, 1.4-3.1)—Fig. 4—Differences in PFS outcomes were neither observed among patients receiving post-infusional IL2 (HR 1.08; 95% CI, 0.87-1.32, *P* = .68) nor regarding administration of prior NMA (HR 1.15; 95% CI, 0.82-1.62; *P* = .41)—Supplementary Material S6. However, a tendency toward higher PFS was demonstrated among patients treated with cancer/testis targeting TCR-T (HR 0.91 95% CI, 0.64-1.3, *P* = .61) and, among these, patients treated with NYESO-1 targeting TCR therapy showed a significantly higher PFS compared to those treated with melanoma differentiation antigens or MAGE-A3 (HR 0.63 95% CI, 0.64-0.98, *P* = .03)—Supplementary Material S6. Such benefit was not demonstrated in terms of DoR, neither among those receiving cancer/testis antigens targeting TCR-T, nor among those specifically treated with NYESO-1 targeting cells. Thus, NYESO-1-based therapies may significantly impact on PFS, whereas DoR seems not to be robustly associated with the type of targeted TAA, but more specifically with the type of response itself. This fact may be related to either tumor or patients associated factors—SuppLementary Material S7.

Duration of response (DoR) was 6.8 months (95% CI, 4.1-11.1) for PR. Median DoR was not reached in patients presenting with CR (overall DoR for CR NR; 95% CI, 24.1-NR). Among patients showing SD, the duration of stability (DoS) was found to be 4.9 months (95% CI, 3.6-7). Differences between DoR and DoS results were significant (log rank *P* < .001)—Fig. 4. CR showed a significantly higher PFS compared to PR cases (HR 0.14; 95% CI, 0.04-0.47; *P* = .002) as well as to SD patients (HR 0.09; 95% CI, 0.02-0.33; *P* < .001). DoR among CR patients at 24 months follow-up was 78%.

No significant differences were noted in terms of DoR or DoS among responders and stabilizers when comparing with either post-infusional IL2 (log rank *P* = .56), prior NMA administration (log rank *P* = .82) or targeted TAA (log rank *P* = .13)—Supplementary Material S8.

### Clinical Predictive Factors for Tumor Response

We sought clinical predictive factors associated with tumor response among patients treated with TCR-based ACT models. To this matter, logistic regression models revealed that infusion of more than 3.9 × 10^9^ cells was associated with a significantly higher probability of tumor response after adjusting for several confounders (OR 6.61; 95% CI, 1.68-21.6; *P* = .007). Similarly, among selected TAAs for TCR production, the analysis revealed that NYESO-1-based TCR therapy was significantly associated with a 3-fold increase of tumor response probability (OR 3.1; 95% CI, 1.18-8.17; *P* = .02). On the contrary, visceral involvement was significantly associated to a decreased likelihood of tumor response (OR 0.25; 95% CI, 0.11-0.56; *P* = .001). Heavily pretreated patients did not show any significant difference in terms of tumor response likelihood in multivariate model. Both, post-infusional IL2 as well as NMA administration showed a non-significant trend toward an increased response likelihood which was not further confirmed after adjusting for multiple confounders in the multivariate model. Results are shown in Table 2.

**Table 2.** Predictive factors associated with tumor response. Right column shows the results of the univariate analysis. Left column shows the results for a multivariate adjusted logistic regression.

	Univariate analysis for ORR likelihood			Multivariate logistic regression for ORR		
	**OR**	**CI 95%**	***P***	**OR**	**CI 95%**	***P***
Age, >50 years^a^	1.33	0.67-2.6	.41	1.02	0.98-1.07	.12
Sex, female = 1^a^	0.49	0.27-0.9	.02^*^	0.51	0.23-1.12	.09
Previous therapy						
Treatment naïve^a^	0.7	0.25-1.98	.49	1.31	0.36-4.73	.68
Previous CHT^b^	0.73	0.37-1.45	.37	0.76	0.134-1.7	.5
Previous ICIs^b^	0.17	0.22-1.32	.09	0.16	0.02-1.56	.12
Previous BioCHT^b^	2	0.82-4.9	.13	1.05	0.27-4.14	.94
Stage						
Visceral involvement^a^	0.24	0.12-0.47	>.001^**^	0.25	0.11-0.56	.001^**^
Brain metastasis^a^	0.81	0.22-3.1	.76	2.51	0.52-12.16	.25
No. of cells. (>3.9 × 10^9^)^*a^	2.32	1.17-4.58	.01^*^	6.61	1.68-26.1	.007^**^
Lymphodeplection, yes = 1^a^	1.25	0.64-2.44	.51	0.67	0.19-2.3	.53
IL2 dosage, LD/HD = 1 vs no = 0^a^	1.3	0.6-2.8	.5	0.5	0.13-1.97	.32
Tumor antigen						
NYESO^c^	3.86	1.73-8.6	.001^**^	3.1	1.18-8.17	.02^*^
MART1^c^	0.65	0.34-1.26	.2	0.49	0.2-1.23	.13
MAGE-A3^c^	0.85	0.32-2.27	.75	1.63	0.43-6.11	.46
gp100^c^	0.62	0.19-1.94	.41	0.39	0.11-1.46	.17

^a^Adjusted by age, sex, treatment naive, visceral involvement, brain metastasis, lymphodeplection, IL2 dosage, number of cells.

^b^Adjusted by age, sex, previous CHT, previous iPD1/iCTLA4, previous BioCHT, visceral involvement, brain metastasis, lymphodeplection, IL2 dosage, number of cells.

^c^Adjusted by age, sex, treatment naive, visceral involvement, brain metastasis, lymphodeplection, IL2 dosage, number of cells.

## Discussion

In this work, we aimed to estimate the efficacy of the use of engineered TCR-T in the therapeutic landscape of cutaneous melanoma. The goal of refining novel ACT strategies is to mainly overcome diverse limitations underlying the specific nature of each modality which are principally summarized in specificity, clonality, and long-lasting anti-tumor cytotoxicity. TCR-based modalities show advantages since they conceptually exert on-tumor cytotoxicity in a monoclonal fashion directed against a pre-specified TAA, with the eventual ability to produce antitumor immune memory.

We hereby present the results of a meta-analysis aiming to address this clinical question, in which we demonstrated an ORR of up to 28% with further DCR reaching to 38%. We decided to include previously conducted trials, regardless of considered key clinical conditions, including both, supportive post-infusional IL2 administration as well as pre-infusional lymphodepletion (NMA). Patients who received post-infusional high-dose IL2 showed higher overall ORR of up to 35% in contrast to those patients who did not receive IL2, among whom ORR was found to be 20%. These results are in line with what was expected based on the studies with ACT utilizing TILs. Similarly, patients who received pre-infusional NMA showed an overall ORR of 35% in contrast to that of 22% among patients who did not receive prior NMA. Nevertheless, even if unadjusted pool estimations showed a trend toward better ORR outcomes among patients who received either NMA or post-infusional IL2, multivariate model did not demonstrate any higher likelihood of tumor response in these subsets. As discussed below, this might result from the wide heterogeneity of the studies included as well as because of lack of statistical power to detect such differences within the pooled cohort.

These results coming from highly selected, mostly HLA-A*0201 positive metastatic cutaneous melanoma patients compare well to the ORR of up to 35%-43% reported in a recent meta-analysis^26^ on metastatic cutaneous melanoma patients treated with NMA, infusion of TILs followed by post-infusional HD IL2. Conceptually, TIL-based therapies may have several advantages in terms of antitumor activity compared to TCR T cells. TIL represents tumor-resident immune cells with a naturally formed polyclonal anti-melanoma TCR repertoire oftentimes targeting more than a single TAA.^27^ In addition, the response, often directed at neoantigens, which result from tumor-specific UV radiation-induced mutations completely foreign to the immune system, may be qualitatively superior to a response against a shared TAA.^28^ Targeting more than one antigen may also lower the chance for immune escape by antigen loss. However, TIL culture takes 3-6 weeks in general, and may consist of a large fraction of terminally differentiated effector T cells, with only a short in vivo half-life, and the fraction of tumor-reactive T cells within TIL may vary widely between patients. In contrast, TCR gene-modified T cells are blood-derived, cultured ex vivo short-term, and may have a much less differentiated phenotype, expressing a high-affinity TCR.

In this analysis, we found a wide range of responses depending on the target, against which the TCR was directed, showing the best overall ORR of up to 51% in patients who received NYESO-1 targeting TCR T cells. In addition, DCR among patients treated with either MART1 or MAGE-A3 targeting TCR-T was remarkably high (up to 37% and 39%, respectively), considering that many of these patients were heavily pretreated. In fact, we conducted a sensitivity analysis, excluding melanoma differentiation antigens, that showed an ORR of up to 40% and a DCR of 45% for cancer/testis antigens-directed TCR-T. Such difference in terms of efficacy among melanoma differentiation antigens and cancer/testis antigens, and specifically that related to NYESO-1 targeting TCR-T is highly remarkable. One of the reasons may be that the retroviral techniques used to produce NYESO-1 targeting TCR-T may have favored a highly monoclonal infusion product, which may in turn show a higher efficacy compared to less fashionable producing strategies such as coculturing immune cells with target antigens among others. Nevertheless, the reason for such differences remains elusive and further studies might be necessary to elucidate our results. Importantly, our efficacy results are very similar to those described for TILs. Therefore, TCR-based therapy may, in selected melanoma patients, already be as effective as that reported for TILs.

Moreover, TCR gene therapies may also yield significant benefits in survival even among pretreated patients. Overall, the impact on PFS seems to be modest, reaching up to 3 months, but for TCR-T therapy targeting cancer/testis antigens a trend toward longer PFS compared to melanoma differentiation antigens is seen. Especially for patients receiving NYESO-1 targeting TCR-T cells, the pooled estimate demonstrates a significantly longer PFS. Interestingly, we found significant differences in duration of response and stabilization. For patients with a complete response the median DoR was not reached, with up to a 78% of complete responders not having progressed after 2 years of follow-up. Patients with partial response as well as stable disease showed longer duration of response/stabilization than patients with progressive disease at first tumor evaluation. These data suggest that TCR-based ACT may still be effective in heavily pretreated metastatic melanoma patients and complete responders may benefit long term, results that are very comparable to what has been reported for CR patients upon TIL therapy.^26^ This is very promising as targeting a single peptide-MHC complex instead of multiple antigens (TIL) can induce long-lasting complete remissions, apparently without immune escape. Obviously and illustrated in this meta-analysis, target selection is key, but perhaps also a better functionality of short-term cultured peripheral blood T cells may impact the outcome. Given the flexibility of current gene editing possibilities, TCR-T-based platforms can provide a wide variety of therapeutic options depending among others on antigen-targeting, costimulatory reinforcement, or checkpoint knockout.^7^

In this study, we also searched for potential predictive factors associated with objective tumor response for which we performed a multivariate adjusted binary regression. Our analysis confirmed that the number of cells infused was significantly associated with probability of tumor response. Correlation between response and number of cells infused has also been demonstrated for TIL therapy.^26^ Moreover, we did not find differences in objective response for pretreated or treatment naïve patients, and prior treatment with immune checkpoint blockade (ICB) or not otherwise specified immunotherapeutic approaches, which suggests that ACT with TCR-T may serve as a valuable treatment option in ICB refractory metastatic melanoma patients. Additionally, NYESO-1 proved to be an independent predictive factor for tumor response confirming our results observed in the pooled estimates for ORR.

Our work has various limitations which need to be further addressed. For instance, due to our unrestricted search strategy the studies included were heterogeneous, not only from a statistical perspective but also because they included a wide variety of TAAs targeting TCR-T cells, different production strategies, different post-infusional IL2 administration dosages as well as a variety of pre-conditioning NMA regimens. In addition, the limited pooled sample size of 187 patients may have been insufficient to reach firm conclusions due to lack of statistical power to identify underlying differences. As an example, we found that ACT-related aspects such as prior lymphodepletion or post-infusional IL2 administration, important for effective cell engraftment, expansion, and persistence, did not show a significant relation with tumor response. Also, because of our limited sample size and the lack of consistent information, a robust conclusion about the relationship between the administration of previous immune checkpoint blockade and the tumor response probability could not be drawn. In contrast, our work was able to firmly identify some interesting factors, which were reproducible even after adjusting for several confounders. These include a correlation between TCR therapy targeting cancer/testis antigens, and especially NYESO-1, and high ORR and survival, which appear comparable to results observed upon TIL therapy, as well as the association between a high number of infused cells with the significantly higher response probability. These factors are of importance for future TCR-T study design.

## Conclusions

ACT with TCR gene-modified T cells has demonstrated benefits in terms of efficacy and survival for patients with metastatic melanoma, even when heavily pretreated. These results show comparability to those reported for TIL-based therapies with respect to ORR and survival, especially with TCR-T targeting cancer/testis antigens and in particular NYESO-1. The apparent advantages of TCR-T-based strategies over TIL treatment coming with rapid developments in gene engineering, create huge potentials, and promises in the field of solid cancers, not only in metastatic melanoma but also beyond.

## Supplementary Material

oyad078_suppl_Supplementary_Material

## Data Availability

The data underlying this article will be shared on reasonable request to the corresponding author.

## Abbreviations

ACT: adoptive cell therapy
CAR: chimeric antigen receptor
CAR-T: chimeric antigen receptor T cell
CR: complete response
DC: dendritic cell
DCR: disease control rate
DoR: duration of response
DoS: duration of stability
FEM: fixed effect model
gp100: glycoprotein 100
HD: high-dose
HLA: human leukocyte antigen
HR: hazard ratio
ICB: immune checkpoint blockade
iCTLA-4: cytotoxic T-lymphocyte associated protein 4 inhibitor
IL2: interleukin 2
iPD1: programmed death 1 inhibitor
LD: low-dose
MAGE-A3: melanoma-associated antigen 3
MART1: melan-A; melanoma antigen recognized by T cells
MHC: major histocompatibility complex
NMA: non-myeloablative
NYESO-1: New York esophageal squamous cell carcinoma 1
OR: odds ratio
ORR: objective response rate
OS: overall survival
PD: progression disease
PFS: progression-free survival
PR: partial response
REM: random effect model
scFv: single chain variable fraction
SD: stable disease
SMAC: supramolecular activation cluster
TAA: tumor associated antigen
TCR: T-cell receptor
TCR-T: T-cell receptor T cell
TIL: tumor-infiltrating lymphocyte
