# Supplementary material for: Efficacy of T-Cell Receptor-Based Adoptive Cell Therapy in Cutaneous Melanoma: A Meta-Analysis
Source: Oncologist. 2023 Apr 10;28(6):e406–15. doi: 10.1093/oncolo/oyad078 (PMC10243774; doi:10.1093/oncolo/oyad078)

Supplementary material S1

	Phase	Lymphodeplection	IL2 dosage	Tumor Antigen	Restriction	Cell product	TCR production	Cell delivery
**Chodon T et al. 2014 [12]**	II	CF 60 + FLU 25	HD	MART1	HLA A*0201	Peripheral T cells	Retroviral transduction	intravenous
**Dréno B et al. 2021 [13]**	I/II	None	LD_a	MART1/MELOE	HLA A*0201	Peripheral T cells	Cocultured with Ag and FACS sorted	intravenous
**Duval L et al. 2015 [18]**	I	None	None	MART1	HLA A*0201	T cell line Ccure 709	Retroviral transduction	intratumoral
**Fontana R et al. 2009 [19]**	I	None	None	MAGE-A3	HLA A*0201	Peripheral T cells	Retroviral transduction	intravenous
**Johnson LA et al. 2009/1 [20]**	II	CF 60 + FLU 25	HD	Cohort 1: MART1<br>Cohort 2: gp100	HLA A*0201	Peripheral T cells	Retroviral transduction	intravenous
**Khammari A et al. 2009 [21]**	II	None	LD_b	MART1	HLA A*0201	Peripheral T cells	Cocultured with Ag	intravenous
**Lu YC et al. 2017 [22]**	Ib	CF 60 + FLU 25	HD	MAGE-A3	HLA DP-B1*0401	Peripheral T cells/CD4+ fraction	Retroviral transduction	intravenous
**Mackensen A et al. 2006 [23]**	I	None	LD_c	MART1	HLA A*0201	Peripheral T cells/CD8+ fraction	Cocultured with Ag-pulsed DC	intravenous
**Mitchell MS et al. 2002 [24]**	I	None	None	Tyrosinase	HLA A*0201	Peripheral T cells/CD8+ fraction	Cocultured with mononuclear as APCs	intravenous
**Morgan RA et al. 2006 [25]**	I	CF 60 + FLU 25	HD	MART1	HLA A*0201	Peripheral T cells/CD8+ fraction	Retroviral transduction	intravenous
**Morgan 2013 RA et al. 2013 [14]**	II	CF 60 + FLU 25	HD	MAGE-A3	HLA A*0201	Peripheral T cells/CD8+ fraction	Retroviral transduction	intravenous
**Robbins PF et al. 2011 [16]**	I	CF 60 + FLU 25	HD	NYESO	HLA A*0201	Peripheral T cells/CD3+ fraction	Retroviral transduction	intravenous
**Robbins PF et al. 2015 [15]**	II	CF 60 + FLU 25	HD	NYESO	HLA A*0201	Peripheral T cells/CD3+ fraction	Retroviral transduction	intravenous
**Yee C et al. 2002/1 [17]**	I	None	LD^d	Cohort 1: MART1<br>Cohort 2: gp100	HLA A*0201	Peripheral T cells/CD8+ fraction	Cocultured with immunogenic epitopes	intravenous

Lymphodeplection: CF 60 + FLU 25 [Cyclophosphamide 60 mg/kg for 2 days followed bu Fludarabine 25 mg/m2 for 5 days]; **IL2 dosage**: HD [720.000 IU/KG q8h to tolerance]; LD [any IL2 dose below that for HD]: a) IL2 s.c. delivered at 6MU from D1-5; b) IL2 s.c. delivered 9MUx3 D1-5 and D8-12 together with s.c. IFN at 9MUx3 during 4 weeks ; c) IL2 s.c. delivered 3MU for 6 days; d) IL2 s.c. delivered at increasing dosage 0,25/0,5/1MU/m2 q12h for 14 days ; **Ag:** Antigen; **DC:** Dendritic Cell; **APC:** Antigen Presenting Cell

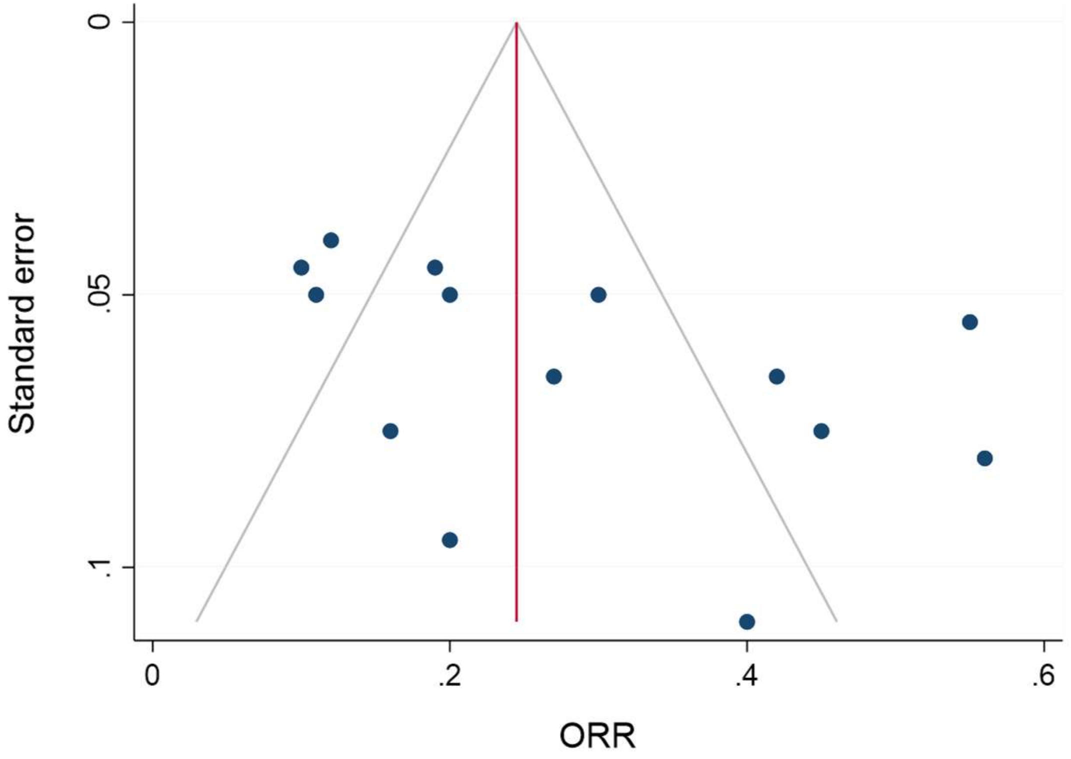

Egger's test p = 0,12

Supplementary material S3

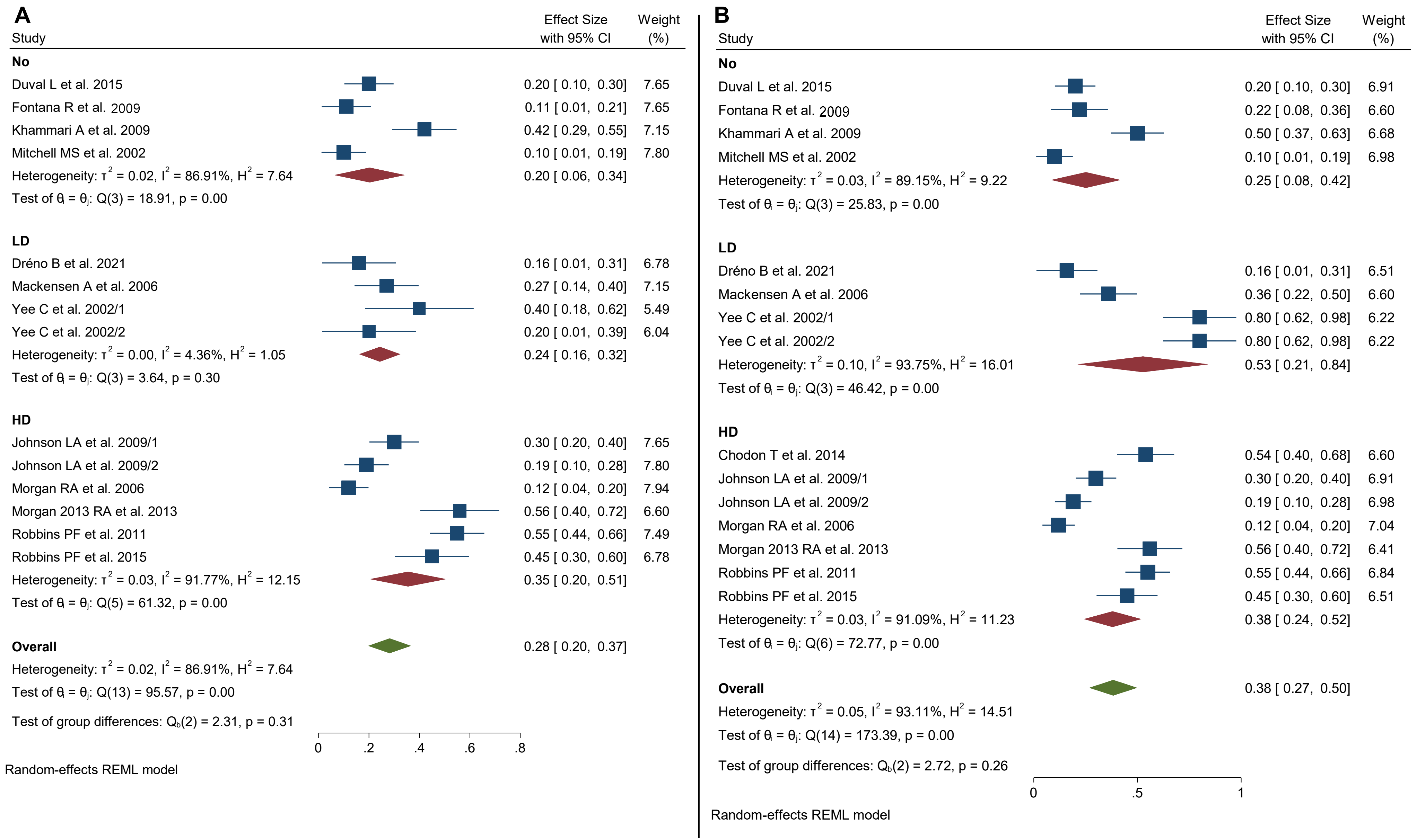

Supplementary material S4

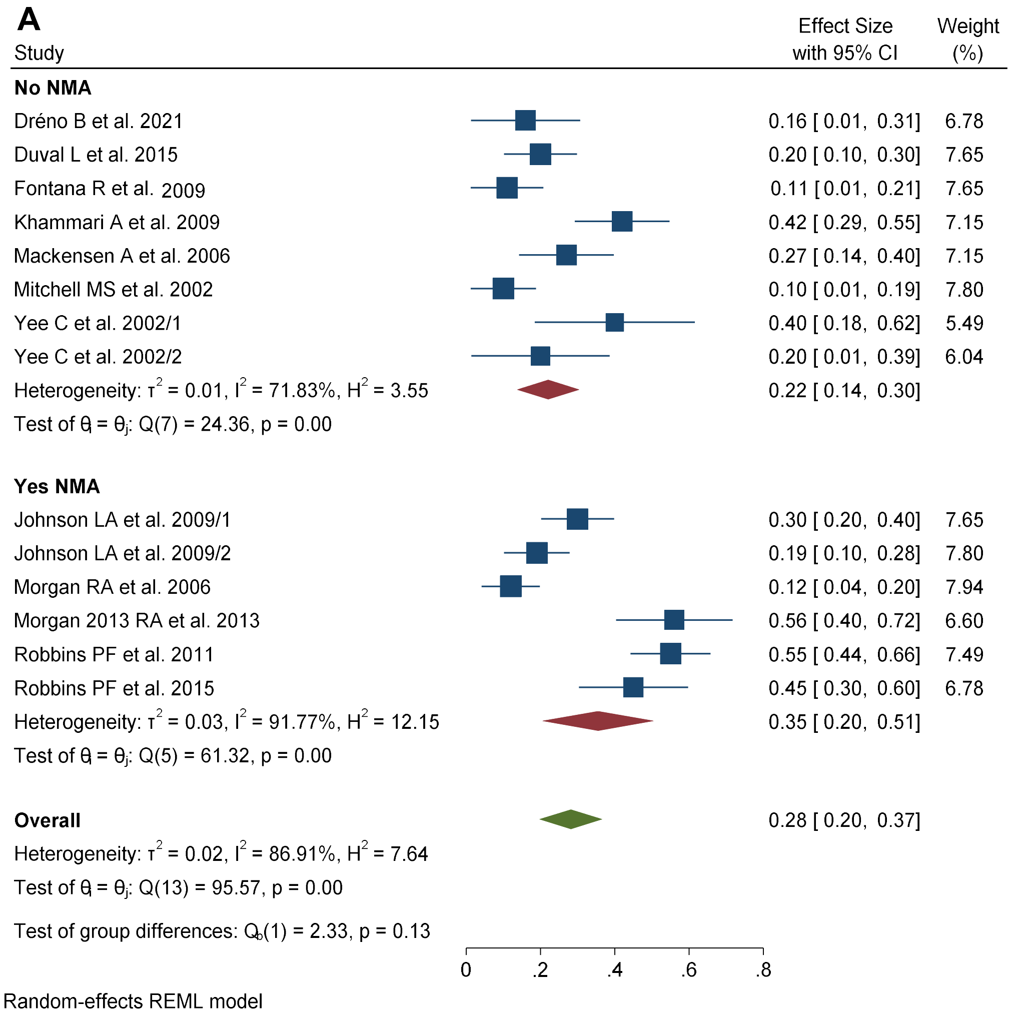

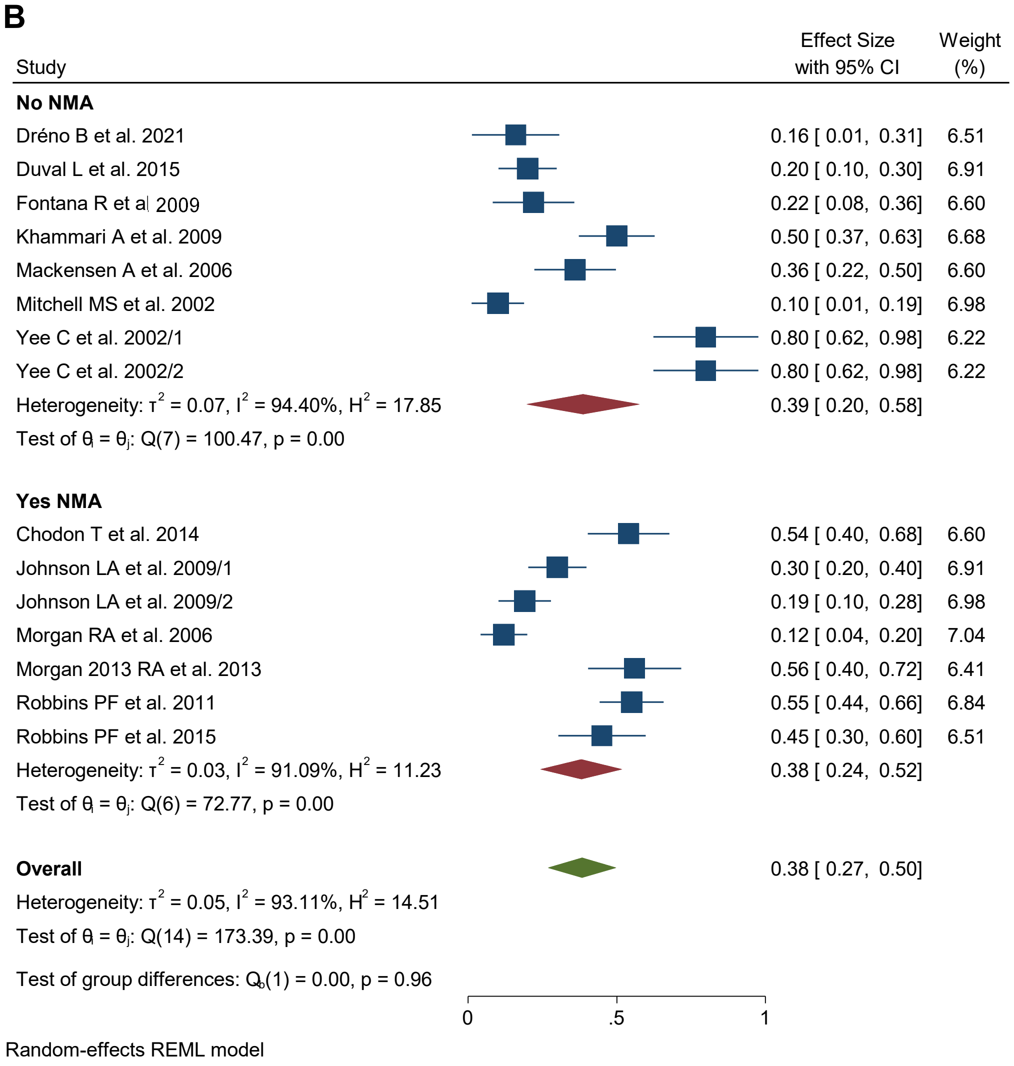

Supplementary material S5

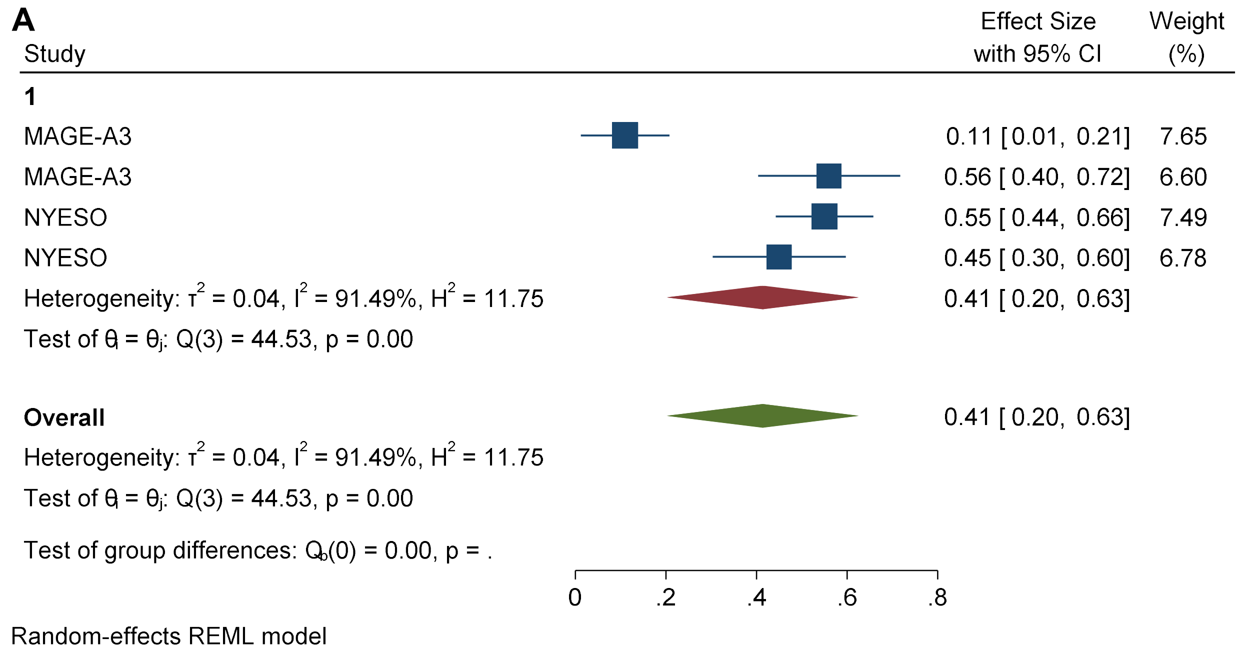

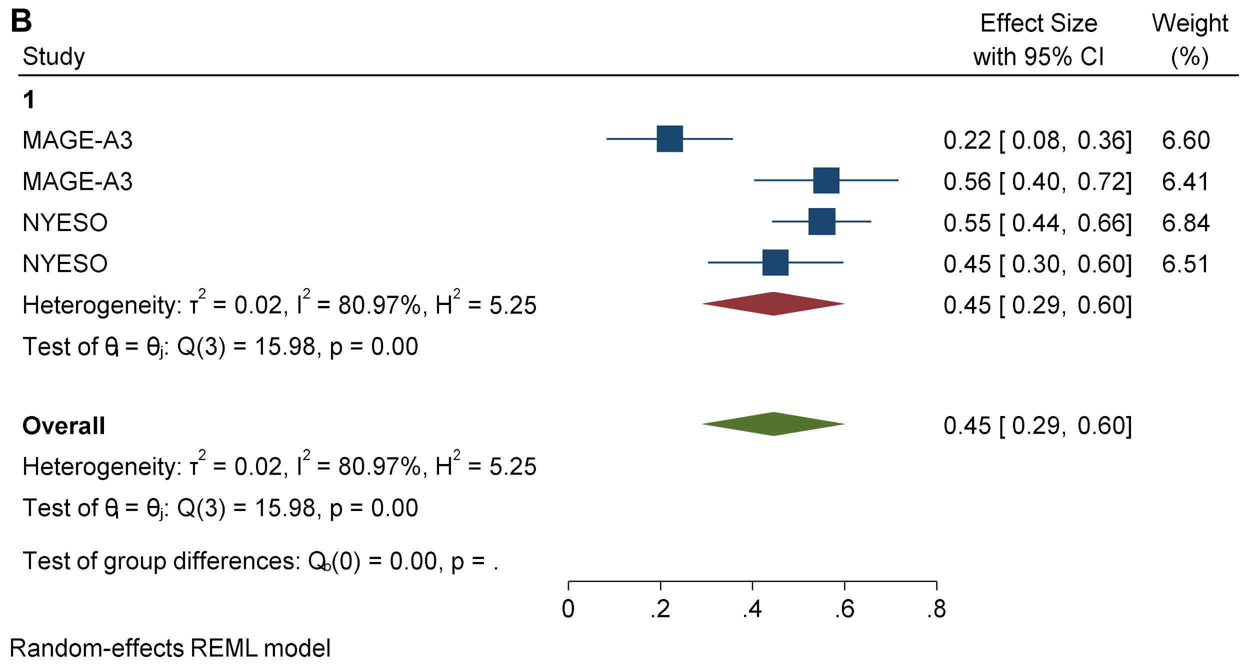

Supplementary material S6

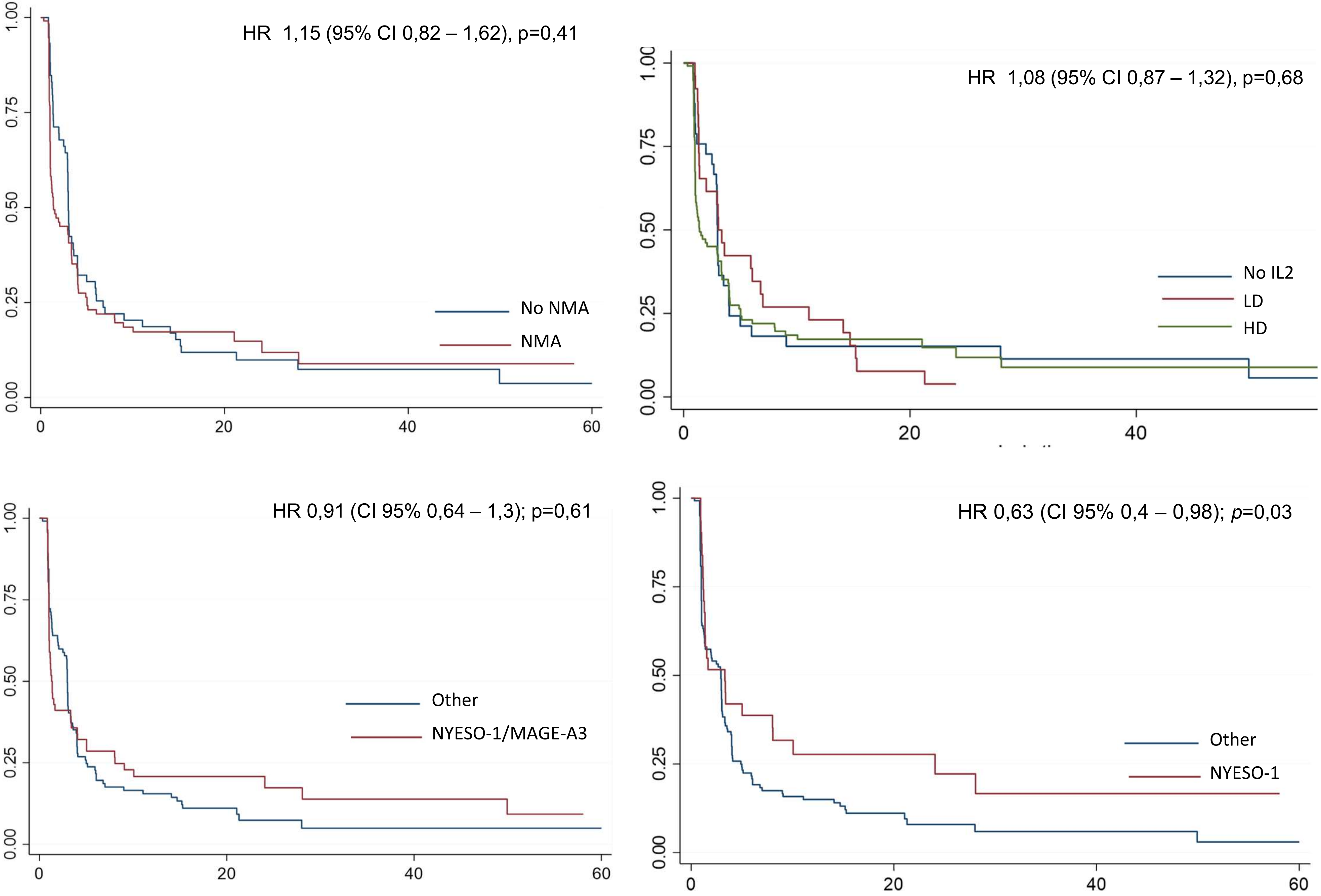

Supplementary material S7

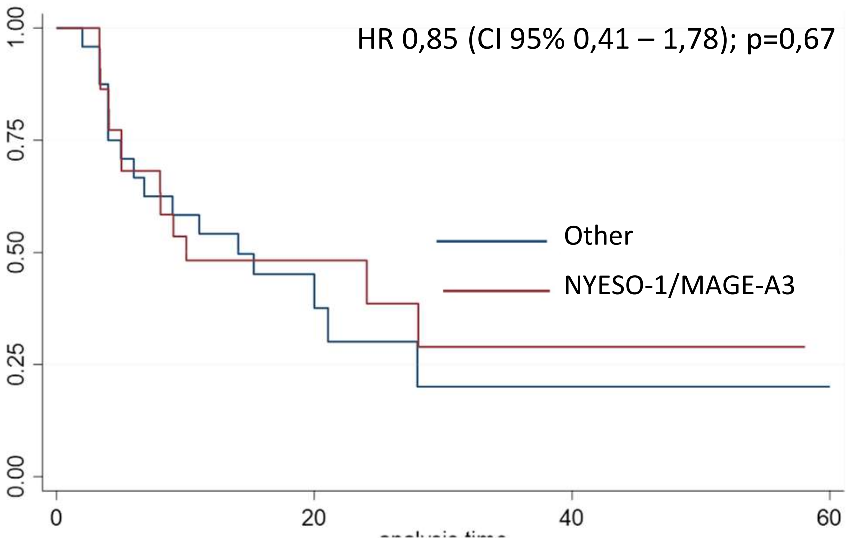

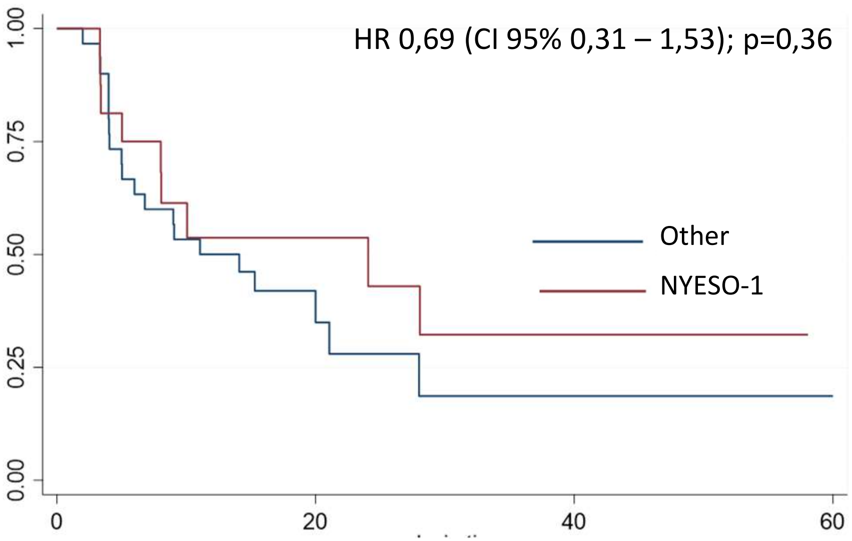

Supplementary material S8

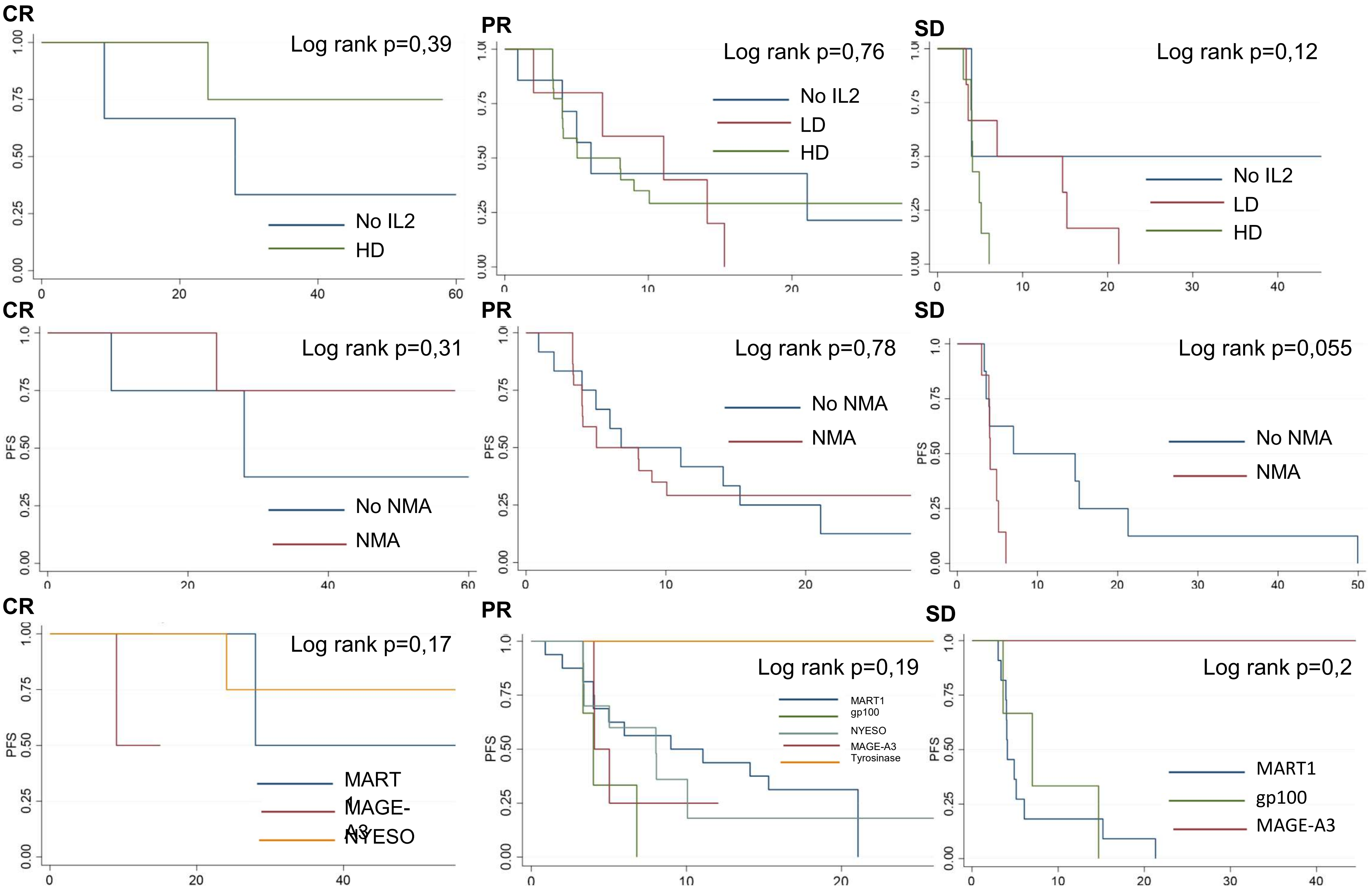

Supplement: oyad078_suppl_Supplementary_Material [file oyad078_suppl_supplementary_material.pdf]
